# The Vast, Conserved Mammalian lincRNome

**DOI:** 10.1371/journal.pcbi.1002917

**Published:** 2013-02-28

**Authors:** David Managadze, Alexander E. Lobkovsky, Yuri I. Wolf, Svetlana A. Shabalina, Igor B. Rogozin, Eugene V. Koonin

**Affiliations:** National Center for Biotechnology Information, National Library of Medicine, National Institutes of Health, Bethesda, Maryland, United States of America; Accelrys, United States of America

## Abstract

We compare the sets of experimentally validated long intergenic non-coding (linc)RNAs from human and mouse and apply a maximum likelihood approach to estimate the total number of lincRNA genes as well as the size of the conserved part of the lincRNome. Under the assumption that the sets of experimentally validated lincRNAs are random samples of the lincRNomes of the corresponding species, we estimate the total lincRNome size at approximately 40,000 to 50,000 species, at least twice the number of protein-coding genes. We further estimate that the fraction of the human and mouse euchromatic genomes encoding lincRNAs is more than twofold greater than the fraction of protein-coding sequences. Although the sequences of most lincRNAs are much less strongly conserved than protein sequences, the extent of orthology between the lincRNomes is unexpectedly high, with 60 to 70% of the lincRNA genes shared between human and mouse. The orthologous mammalian lincRNAs can be predicted to perform equivalent functions; accordingly, it appears likely that thousands of evolutionarily conserved functional roles of lincRNAs remain to be characterized.

## Author Summary

Genome analysis of humans and other mammals reveals a surprisingly small number of protein-coding genes, only slightly over 20,000 (although the diversity of actual proteins is substantially augmented by alternative transcription and alternative splicing). Recent analysis of the mammalian genomes and transcriptomes, in particular, using the RNAseq technology, shows that, in addition to protein-coding genes, mammalian genomes encode many long non-coding RNAs. For some of these transcripts, various regulatory functions have been demonstrated, but on the whole the repertoire of long non-coding RNAs remains poorly characterized. We compared the identified long intergenic non-coding (linc)RNAs from human and mouse, and employed a specially developed statistical technique to estimate the size and evolutionary conservation of the human and mouse lincRNomes. The estimates show that there are at least twice as many human and mouse lincRNAs than there are protein-coding genes. Moreover, about two third of the lincRNA genes appear to be conserved between human and mouse, implying thousands of conserved but still uncharacterized functions.

## Introduction

The great majority of mammalian genome sequences are transcribed, at least occasionally, a phenomenon known as pervasive transcription [1]–[4]. More specifically, tiling array analyses of several human chromosomes have shown that over 90% of the bases are transcribed in at least one cell type [1], [5]–[8]. The analogous analysis in mouse has demonstrated transcription for over 60% of the genome [9]–[11]. Among the transcripts there are numerous long intergenic non-coding RNA (lincRNA), i.e. RNA molecules greater than 200 nucleotides in length that are encoded outside other identified genes. Some of the lincRNAs have been shown to perform various regulatory roles but the majority remain functionally uncharacterized [7], [12]–[17]. Furthermore, the fraction of the genome allotted to lincRNAs remains unknown.

A popular view that the vast majority of lincRNAs are by-products of background transcription, “simply the noise emitted by a busy machine” [18], [19], is rooted in their typically low abundance and poor evolutionary conservation compared to protein-coding sequences and small RNAs such as miRNAs and snoRNAs [20]. However, some of the lincRNAs do contain strongly conserved regions [21], and most lincRNAs show reduced substitution and insertion/deletion rates suggestive of purifying selection [12], [22], [23].

Given the general lack of strong sequence conservation, identification of lincRNAs on genome scale relies on expression analysis which makes comprehensive characterization of the mammalian lincRNome an elusive goal. The combination of different experimental approaches applied to transcriptomes of several species has resulted in continuous discovery of new transcripts [24], with the FANTOM project alone cataloguing more than 30,000 putative long non-coding transcripts in mouse tissues by full-length cDNA cloning [11], [25]. The Support Vector Machine method has been applied to classify transcripts from the FANTOM3 project into coding and non-coding ones and accordingly estimate the number of long non-coding RNA in mouse. This analysis has led to the identification of 14,000 long non-coding RNAs and an estimate of the total number of such RNAs in the FANTOM3 data at approximately 28,000 [26].

Here we re-analyze the most reliable available sets of human and mouse lincRNAs using the latest next generation sequencing (RNAseq) data and apply a maximum likelihood approach to obtain a robust estimate of the size of the mammalian lincRNome. The results suggest that mammalian genomes are likely to encode at least twice as many lincRNAs as proteins.

## Results

### Estimation of the sizes of human and mouse lincRNomes

We performed comparative analysis of the recently reported validated sets of 4662 human lincRNAs [27] and 4156 mouse lincRNAs [12], [20], [23] (see Methods for details) in an attempt to produce robust estimates of the human and mouse lincRNome sizes, and to measure the turnover of lincRNA genes in mammalian evolution. The validated sets consist of lincRNA species for which a specific profile of expression across tissues – and hence distinct functionality – are supported by multiple lines of evidence. Assuming that these sets of lincRNAs are random samples from human and mouse lincRNomes, comparison of the validated sets should yield robust estimates of the lincRNome size for each species. For this analysis, we deliberately chose to employ the validated sets only rather than the available larger sets of reported putative lincRNAs in order to reduce the effect of transcriptional noise and other artifacts.

A substantial fraction of the vast mammalian transcriptome, most likely the lower expressed transcripts, is expected to be non-functional. Therefore, to minimize the contribution of transcriptional noise, cut-off values were imposed on expression levels of lincRNA genes and their putative orthologs that were used for the lincRNome size estimation. Similarly, a series of cut-off values was applied for the fraction of indels in pairwise genomic alignments (see Methods for details).

**Figure 1:**
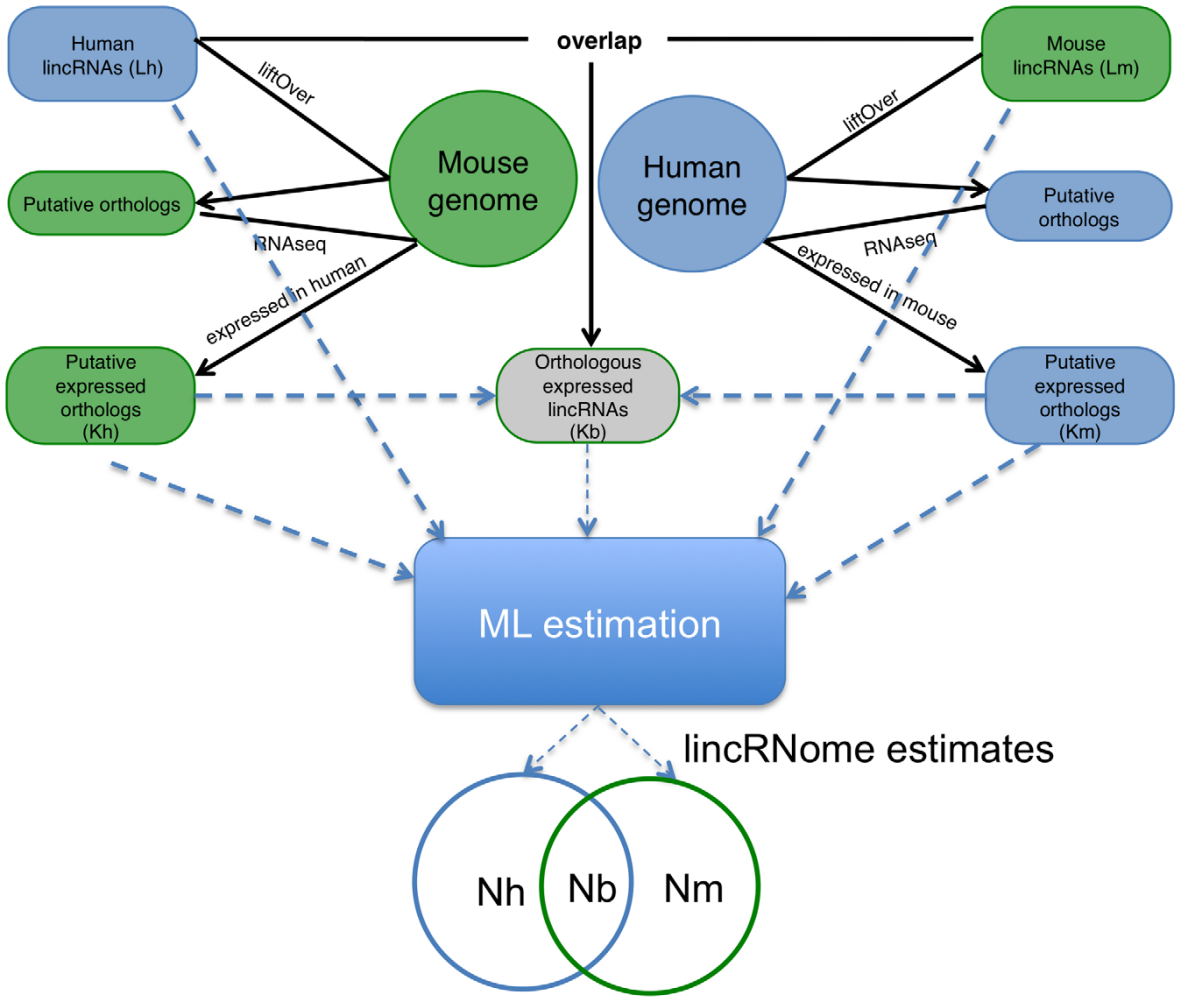
Computational pipeline to characterize the lincRNome. The subset of orthologous lincRNAs (Kb) was obtained by comparing genomic positions of mouse and human lincRNA genes (minimal overlap 100 nucleotides), with further manual inspection of the genomic alignments. This comparison yielded 196 pairs of unique orthologous pairs of human and mouse lincRNA genes (*Kb*). Of the 4662 human lincRNAs (*Lh*), corresponding alignable regions in mouse were detected for 3529. These sequences were designated putative orthologs and checked for evidence of expression using RNAseq data for mouse tissues. Of the 3369 putative lincRNAs, for which the exon models could be determined, 2872 showed expression level greater than zero (*Kh*). Similarly, the subset of mouse lincRNAs with expressed putative orthologs (*Km*) was identified by searching for evidence of expression in human tissues. Of the 4156 mouse lincRNAs (*Lm*), for 3157 corresponding alignable regions with expression level greater than zero were identified in mouse. After applying ORF (<120 nucleotides), indel and expression thresholds (see Methods for details), final results (Figure 2 and Table 1) were obtained using a Maximum Likelihood Model (see Methods for details) and *Lm, Lh, Km, Kh, Kb* as input parameters (shown by dashed arrows) to estimate the size of the human lincRNome (*Nh*), the mouse lincRNome (*Nm*) and the orthologous subset of the two lincRNomes (*Nb*). For details of the procedures see Methods.

A computational pipeline was developed to compare the sets of validated lincRNAs from human and mouse and to identify expressed orthologs by mapping the sequences to the respective counterpart genome and searching the available RNAseq data [28] (Figure 1). We then applied a maximum likelihood (ML) technique to estimate the total number of lincRNA genes in the human and mouse genomes as well as the number of orthologous lincRNA genes (see Online Methods). The following simplifying assumptions were made:

1. A lincRNA sequence in one species has at most one ortholog in the other species (that is lineage-specific duplications are disregarded).
2. The sets of experimentally validated lincRNAs are random samples from complete sets of lincRNAs (lincRNomes) for the corresponding species.
3. The experimentally validated lincRNA sets for human and mouse are uncorrelated with each other.

Let *Lh* and *Lm* be the sizes of the experimentally validated sets of lincRNAs for human and mouse, respectively. Also let *Kh* be the number of confirmed human lincRNAs that have an expressed orthologous sequence in mouse and *Km* be the corresponding number of mouse lincRNAs. Finally, *Kb* is the number of confirmed, expressed human lincRNAs whose orthologs in mouse are also confirmed lincRNAs. If the orthology relations between the human and mouse lincRNAs are strictly one-to-one, the number of confirmed mouse lincRNAs for which the human ortholog is also a confirmed lincRNA should be *Kb* as well. This is indeed the case in practice, with a few exceptions.

Given assumption (1), the lincRNAs can be partitioned into three pools: i) those present in both species, pool size *Nb*, ii) unique to human, *Nh*-*Nb*, and ii) unique to mouse, *Nm*-*Nb*; here *Nh* and *Nm* are the total sizes of the complete human and mouse lincRNomes, respectively. Assumption (2) allows us to compute the probability of observing a particular set of *Kh*, *Km* and *Kb* simply by counting the number of possible samples of *Lh* and *Lm* lincRNAs drawn at random from the respective pools of *Nh* and *Nm* that result in the given set of *Kh*, *Km* and *Kb* values:
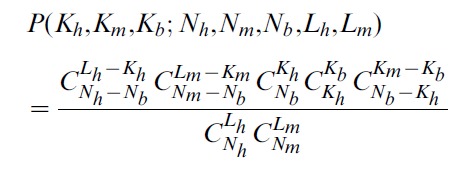
(1)Maximizing the probability P in Eq. (1) with respect to Nh, Nm and Nb, we obtain (see Methods for details):
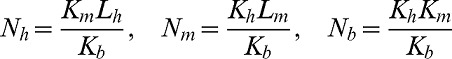
(2)


**Figure 2:**
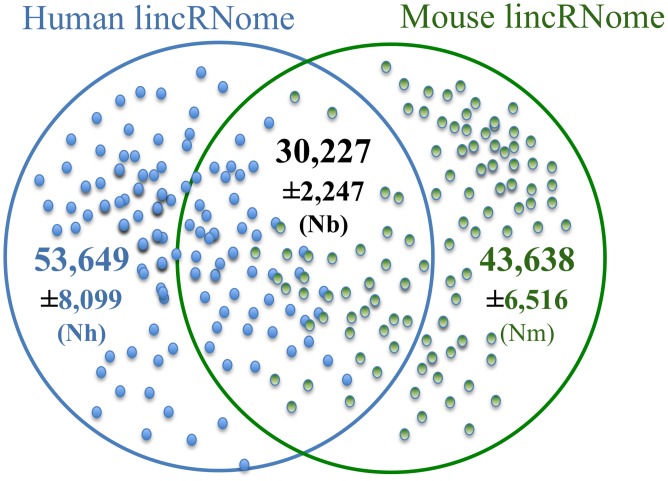
The human and mouse lincRNomes. The figure shows the estimated numbers of lincRNA genes in human and mouse, and the estimate for the size of the set of orthologous lincRNAs. The circles show the estimated sizes of the human and mouse lincRNomes (*Nh* and *Nm*, respectively), and the overlap shows the estimated number of orthologous lincRNAs (*Nb*). For each of these values, the analytically determined 95% confidence intervals are indicated; bootstrap analysis yielded more narrow confidence intervals (see Methods for details). The small, filled circles (blue and green for human and mouse, respectively) show the validated sets of lincRNAs (*Lh* and *Lm*, respectively), and the overlap area between these circles shows orthologous expressed, validated lincRNAs (*Kb*).

To assess the robustness of the estimates, ranges of open reading frame size thresholds used to eliminate putative protein-coding genes and RPKM (reads per kilobase of exon model per million mapped reads [29]) thresholds used to gauge the expression level were employed (Tables 1 and 2). The ML estimates converged at approximately 50,000 lincRNAs encoded in the human genome and approximately 40,000 lincRNAs encoded in the mouse genome (Table 1 and Figure 2). These are conservative estimates given the use of strict thresholds on predicted open reading frame size and expression level (Table 1), so the actual numbers of lincRNAs are expected to be even greater.

**Table 1:** RPKM-based estimates of the numbers of all and orthologous lincRNAs.

	ORF thresholds			
	<90 nt	<120 nt	<150 nt	None
**Lh**	2,961	3,603	3,989	4,662
**Lm**	2,806	3,332	3,644	4,156
**Kh**	1,655	2,030	2,249	2,641
**Km**	1,947	2,308	2,531	2,888
**Kb**	130	155	170	196
**Nh**	**44,346**±7308	**53,649**±8099	**59,389**±8563	**68,693**±9224
**Nm**	**35,722**±5817	**43,638**±6516	**48,207**±6876	**55,999**±7444
**Nb**	**24,786**±4007	**30,227**±4481	**33,483**±4741	**38,914**±5135
**%conservation(human/mouse)**	**56/69**	**56/69**	**56/69**	**57/69**

Two expression and four indel thresholds were applied to putative orthologous lincRNA genes (Kh and Km).

**Table 2:** Estimates of the numbers of all and orthologous lincRNAs with varying expression thresholds^a^.

	Expression thresholds (RPKM)								
	90%	80%	70%	60%	50%	40%	30%	20%	10%
**Lh**	3141	2792	2443	2094	1745	1396	1047	698	349
**Lm**	2530	2249	1967	1686	1405	1124	844	562	281
**Kh**	1819	1638	1433	1242	1042	842	640	427	217
**Km**	1838	1653	1451	1242	1050	847	646	437	226
**Kb**	145	139	130	117	96	80	72	49	25
**Nh**	**42,458**	**39,814**	**33,202**	**27,267**	**22,228**	**19,085**	**14,780**	**9,393**	**6,225**
**Nm**	**33,719**	**31,738**	**26,502**	**21,682**	**17,897**	**15,250**	**11,830**	**7,502**	**4,897**
**Nb**	**24,424**	**23,057**	**19,479**	**15,994**	**13,184**	**11,396**	**8,914**	**5,742**	**3,808**
**%**	**58/72**	**58/73**	**59/74**	**59/74**	**59/74**	**60/75**	**60/75**	**61/77**	**61/78**

a Indel threshold: 95%, ORF threshold: 120 nt (see Methods). Expression thresholds were applied to lincRNA genes (Lh, Lm, and Kb) and putative orthologous lincRNA genes (Kh and Km).

% stands for conservation percentage as in Table 1.

Approximately two-thirds of the lincRNA genes were estimated to share orthologous relationships (Figure 2 and Table 1). The subsets of lincRNAs with the increasing expression levels were found to be smaller and slightly but consistently more conserved (Table 2), a result that is compatible with our previous observation of positive correlation between sequence conservation and expression level among lincRNAs [23].

We next used the length distributions of human and mouse lincRNAs in the validated sets to estimate the total lengths of the lincRNomes and the fraction of the genome occupied by the lincRNA-encoding sequences, once again under the assumption that the validated sets are representative of the entire lincRNomes. Strikingly, the fraction of the human and mouse euchromatic genome sequence dedicated to encoding lincRNAs was found to be more than twofold greater than the fraction allotted to protein-coding sequences and greater even than the total fraction encoding mRNAs (including untranslated regions) (Table 3).

**Table 3:** The fractions of the human and mouse genomes allotted to protein-coding and lincRNA-coding sequences^a^.

	Human	Mouse
**Protein-coding genes, number**	19,042	20,210
**Protein-coding sequences, total length (Mb)/% genome**	32.6/1.23	33.5/1.27
**UTRs, total length (Mb)/% genome**	21/0.79	19/0.72
**lincRNAs, validated set, number**	4,662	4,156
**lincRNAs, validated set, total length (Mb)**	6.2^b^	7.8
**lincRNAs, genomic estimate, number**	53,649	43,638
**lincRNAs, genomic estimate, total length (Mb)/%genome**	72.5/2.7	81.9/3.1

a The data on protein-coding genes and the total size of the euchromatic genomes are from [31], [63].

b The total length of the human lincRNome is likely to be an underestimate caused by the use of RNAseq data to calculate the lengths of lincRNAs in the human validated set [27].

## Discussion

The relatively poor sequence conservation and often low expression of lincRNAs hamper robust estimation of the size of the lincRNome from expression data alone and render comparative-genomic estimation an essential complementary approach. Strikingly, the estimates obtained here by combining comparative genomic and expression analysis suggest that the mammalian lincRNome is at least twice the size of the proteome [30], [31]. Given that intron-encoded long-non-coding RNAs and non-coding RNAs encoded in complementary strands of protein-coding genes (long antisense RNAs) [32] are disregarded in these estimates, the total set of lncRNAs and the fraction of the genome dedicated to the lincRNA genes are likely to exceed the respective values for protein-coding genes several-fold.

In order to assess the reliability and robustness of the model with respect to parameters, we produced series of estimates of the total size of the human and mouse lincRNomes and their conserved subset with varying thresholds on expression level, extent of sequence similarity and the maximum allowed open reading frame size. Nevertheless, it is impossible to rule out some sources of bias that might have affected the estimates. For example, some orthologous lincRNA genes might remain undetected because they were not included in the UCSC genome alignments due to high divergence or synteny breaks in (for example, inversions or translocations). Such under-detection of orthologs could cause an underestimate of evolutionary conserved lincRNA genes although it has been reported that the of breakpoints is not large (<250) for the human/mouse genomic comparison [33], so this type of bias is likely to be negligible. Another, potentially more serious source of bias could be a correlation between two lists of lincRNA genes which again would result in biased estimates of evolutionary conserved lincRNA genes. However, because the human and mouse lincRNA sets were obtained using quite different approaches [12], [20], [23], [27], there is no reason to expect that any strong correlation between the two lists would be caused by the employed experimental and/or computational procedures. An under-estimate of the number of orthologous lincRNAs as well as the total size of the mouse lincRNome also might be caused by smaller RNAseq dataset for mouse (10 tissue/cell types, see Methods for details) compared to human (16 tissue/cell types). This difference could explain the systematically smaller predicted numbers of mouse lincRNA genes (Tables 1 and 2). More generally, given that expression of a large fraction of lincRNAs appears to be tissue-specific, the availability of sufficient data for relatively small numbers of tissue/cell types could cause substantial underestimate of the size of both lincRNomes and their conserved fraction. Thus, the estimates obtained here should be regarded as highly conservative, essentially low bounds the lincRNome size and the set of orthologous lincRNA genes.

Some of the transcripts identified as lincRNAs potentially might represent fragments generated from long (alternative) 5′UTRs or 3′UTRs of protein-coding genes. Such transcripts could results from utilization of alternative poly(A) addition signals and/or could represent alternative splice forms separated by long introns [3], [18], [19], [34]. If many purported lincRNAs actually are fragments of protein-coding genes, one would expect a strong correlation to exist between the expression of lincRNAs and neighboring protein-coding genes. Cabili and co-workers analyzed this correlation for the set of validated human lincRNA genes [27]. Their analysis focused on those protein-coding genes that had a lincRNA neighbor on one side and a coding neighbor on the other side, and used a paired test to compare the correlation between each protein-coding gene and its lincRNA neighbor with that between the same protein-coding gene and its protein-coding gene neighbor. This comparison showed a weak opposite trend, namely that expression of pairs of coding gene neighbors was, on average, slightly but significantly more strongly correlated than the expression of neighboring lincRNA/protein-coding gene pairs. The results of this analysis appear to be best compatible with the hypothesis that any co-expression between lincRNAs and their protein-coding neighbors results from proximal transcriptional activity in the surrounding open chromatin [35]. These findings effectively rule out the possibility that the majority of lincRNAs are fragments of neighboring protein-coding genes although there are anecdotal observations that 3′UTR-derived RNAs can function not only in *cis* to regulate protein expression but also intrinsically and independently in *trans*, likely as non-coding RNAs [36].

The possibility that some lincRNA genes encode short peptides that are translated, perhaps in a tissue-specific manner, is the subject of an ongoing debate [13], [37]–[40]. It is extremely hard to rule out such a role for a fraction of purported lincRNAs as becomes obvious from the long-standing attempts to investigate potential functions of the thousands upstream open reading frames (uORFs) that are present in 5′UTR of protein-coding genes in eukaryotes [41]–[44]. Although some of the uORFs are translated, the functions of the produced peptides if any remain unclear [45]. Even application of modern high-throughput techniques in simple eukaryotic model systems so far have failed to clarify this issue. For example, analysis of 1048 uORFs in yeast genes has supported translation of 153 uORFs [46]. Furthermore, numerous uORF translation start sites were found at non-AUG codons, the frequency of these events was even higher than for uAUG codons even though the frequency of non-AUG starting codons is extremely low for protein-coding genes [46]. Another intriguing recent discovery is the potential presence, in the yeast genome, of hundreds of transiently expressed ‘proto-genes’ that are suspected to reflect the process of *de novo* gene birth [40]. However, the functionality of these peptides remains an open question. Establishing functionality of short ORFs in mammalian genomes is an even more difficult task. For example, analysis of translation in mouse embryonic stem cells revealed thousands of currently unannotated translation products. These include amino-terminal extensions and truncations and uORFs with regulatory potential, initiated at both AUG and non-AUG codons, whose translation changes after differentiation [47]. However, contrary to these emerging indications of abundant production of short peptides, a recent genome-wide study has reported very limited translation of lincRNAs in two human cell lines [48]. In general, at present it appears virtually impossible to annotate an RNA unequivocally as protein-coding or noncoding, with overlapping protein-coding and noncoding transcripts further confounding the issue. Indeed, it has been suggested that because some transcripts can function both intrinsically at the RNA level and to encode proteins, the very dichotomy between mRNAs and ncRNAs is false [38].

Taking all these problems into account, here we adopted a simple, conservative approach by excluding from the analysis lincRNAs containing relatively long ORFs, under a series of ORF length thresholds. However, it should be noted that human and mouse lincRNAs used in this study had been previously filtered for the presence of evolutionary conserved ORFs and the presence of protein domains, and the most questionable transcripts were removed at this stage [12], [20], [23], [27]. For example, 2305 human transcripts were excluded from the stringent human lincRNA set [27] under the coding potential criteria (the presence of a Pfam domain, a positive PhyloCSF score, or previously annotated as pseudogenes). The majority of these discarded transcripts (1533) were previously annotated as pseudogenes [27]. Similar to the stringent set of lincRNAs, these transcripts are expressed at lower and more tissue-specific patterns than bona fide protein-coding genes, suggesting that these effectively are non-coding transcripts. Nevertheless, Cabili and co-workers employed a conservative approach and excluded them from the stringent lincRNA set [27].

Questions about functional roles of lincRNAs and the fraction of the lincRNAs that are functional loom large. For a long time, the prevailing view appeared to be that, apart from a few molecular fossils such as rRNA, tRNA and snRNAs, RNAs did not play an important role in extant cells. More recently, the opposite position has become popular, namely that (almost) every detectable RNA molecule is functional. It has been repeatedly pointed out that this view is likely to be too extreme [49], [50]. Although it has been shown that many lincRNA genes are evolutionarily conserved and perform various functions [7], [12]–[17], an unknown fraction of lincRNAs should be expected to result from functionally irrelevant background transcription [19]. In the present work, phylogenetic conservation is the principal support of functional relevance of lincRNAs. Given that neutrally evolving sequences in human and mouse genomes are effectively saturated with mutations and show no significant sequence conservation [51]–[53], expression of non-coding RNAs at orthologous genomic regions in human and mouse should be construed as strong evidence of functionality. It should be noted, however, that sequence conservation gives the low bound for the number of functional lincRNAs, and the lack of conservation is not a reliable indication of lack of function. First, the possibility exists that orthologous genes diverge to the point of being undetectable by sequence comparison, e.g. because short conserved, functionally important stretches are interspersed with longer non-conserved regions, as is the case in Xist, H19, and similar lincRNAs [54], [55] [20].

The results of this work predict that, despite the fact that on average sequence conservation between orthologous lincRNAs is much lower than the conservation between protein-coding genes [12], [23], 60 to 70% of the lincRNAs appear to share orthologous relationship between human and mouse, which is only slightly lower than the fraction of protein-coding genes with orthologs, approximately 80% [51]. These findings imply that, even if many of the species-specific lincRNAs are non-functional, mammalian lincRNAs perform thousands of evolutionarily conserved functional roles most of which remain to be identified.

## Methods

### The human and mouse validated lincRNA sets

As the human lincRNA data set, the ‘stringent set’ of 4662 lincRNAs, which is a subset of the over 8000 human lincRNAs described in a recent comprehensive study [27], was used. The validated set of mouse lincRNA genes was constructed by merging our previously published set of 2390 lincRNA transcripts with the set of 3051 transcripts produced by Ponting and coworkers [12]. After the merge, a unique list of 4989 GenBank transcript IDs was generated, coordinates of the newest mouse assembly, mm9, were downloaded in BED format from the UCSC Table Browser [56], and entries shorter than 200 nt were discarded. Overlapping chromosomal coordinates were merged using the mergeBed utility from BEDtools package [57], with the command line option -s (“force strandedness”, i.e. merge overlapping features only if they are on the same strand), and unique IDs were assigned to the resulting 4156 mouse lincRNA clusters. (format: mlclust_N where mlclust stands for mouse lincRNA cluster, and N is a unique integer number; see Supporting Table S1).

### Expression of lincRNAs

Expression of the lincRNAs was assessed by analysis of the available RNAseq data. For human, the run files of the Illumina Human Body Map 2.0 project for adipose, adrenal, brain, breast, colon, heart, kidney, liver, lung, lymph node, ovary, prostate, skeletal muscle, testis, thyroid, white blood cells, were downloaded from The NCBI Sequence Read Archive (SRA, http://www.ncbi.nlm.nih.gov/Traces/sra; Study ERP000546; runs ERR030888 to ERR030903). For mouse, RNAseq data of the ENCODE project [58] for tissues: bone marrow, cerebellum, cortex, ES-Bruce4, heart, kidney, liver, lung, mouse embryonic fibroblast cells (MEF) and spleen, were downloaded from the UCSC Table Browser [56] FTP site (ftp://hgdownload.cse.ucsc.edu/goldenPath/mm9/encodeDCC/wgEncodeLicrRNAseq/).

Pre-built Bowtie indices of human and mouse, based on UCSC hg19 and mm9, were downloaded from Bowtie FTP site (ftp://ftp.cbcb.umd.edu/pub/data/bowtie_indexes/hg19.ebwt.zip and ftp://ftp.cbcb.umd.edu/pub/data/bowtie_indexes/mm9.ebwt.zip, respectively). The reads were aligned with the cognate genomic sequences using TopHat [59].

The TopHat-generated alignments were analyzed using an ad hoc Python script that accepts alignments and genomic coordinates in SAM and BED formats, respectively, and uses the HTSeq Python package (http://www-huber.embl.de/users/anders/HTSeq) to calculate the number of aligned reads (“counts”). The RPKM (i.e. reads per kilobase of exon model per million mapped reads [29]) values were calculated from the counts values. Because we were interested to determine whether particular regions are expressed in any of the analyzed tissues, the maximum value among all tissues was assigned as the expression level of lincRNA genes and putative orthologous lincRNA genes.

### Identification of open reading frames (ORFs)

An ORF was defined as a continuous stretch of codons starting from the ATG codon or beginning of the cDNA (to take into account potentially truncated cDNAs) and ending with a stop codon. The ORFs were identified by using the ATG_EVALUATOR program [60] combined with the ORF predictor from the GeneBuilder package [61] with relaxed parameters (the program was required to correctly predict 95% of the human and mouse cDNA training sets [61]). Control experiments with independent human and mouse cDNA data sets [61] showed a 94–98% true positive rate depending on the ORF length threshold (90, 120 or 150 nucleotides). However, a high rate of false positives is expected for such relaxed parameters. Analysis of human and mouse introns and UTRs data sets showed false positives rates of 10–20% depending on the threshold [60], [61]. For the purpose of the present analysis, false positives in ORF identification represent random removal of lincRNA sequences from the samples resulting in conservative estimates of the total lincRNA number. Thus, we used the ORF cut-off values of 90, 120 or 150 nucleotides to remove putative mRNAs for short proteins separately from the human and mouse sets of lincRNAs.

### Comparative genomic analysis of the lincRNA sets

To obtain the subset of human lincRNAs with expressed orthologs in mouse (*Kh*), human lincRNA gene coordinates of assembly hg19 were converted to mouse mm9 using the liftOver tool of the UCSC Genome Browser [62]. Out of the 4662 human lincRNAs (*Lh*), for 3529 putative orthologous regions were identified in the mouse genome. These sequences were checked for the evidence of expression in mouse tissues using the RNAseq data. Exon coordinates of putative lincRNAs were obtained by mapping their coordinates onto exons of all known genes of mm9 assembly of UCSC Genome Browser. The sums of exons were then used in expression level calculation to normalize for sequence length. Out of the 3369 putative lincRNAs for which the exon models could be determined, 2872 had expression level greater than zero. Similarly, the subset of mouse lincRNAs with expressed putative orthologs in human (*Km*) was found by converting the coordinates of initial 4156 mouse lincRNAs (Lm) from mm9 to hg19 and searching for the evidence of expression in human tissues. The exon models could be determined for 3656 of the 3677 putative lincRNAs, out of which 3157 had expression level greater than zero.

The subset of orthologous lincRNAs (*Kb*) was obtained by selecting those lincRNAs whose putative orthologs in another species overlap with the validated lincRNAs of that species. That is, we searched for the overlap of putative orthologs of human lincRNAs (in hg19 coordinates) with the mouse lincRNAs (in mm9 coordinates, minimal overlap 100 nucleotides). The overlap was determined using intersectBed from BEDtools package with the command line option -s (“force strandedness”). This resulted in 196 pairs of unique human and mouse lincRNAs. Approximate indel values were estimated from the sequence length differences between the lincRNAs and their orthologs, i.e. the following formula was used:
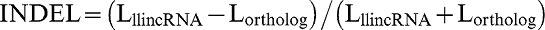
where L_llincRNA_ is the total length of lincRNA exons, and L_ortholog_ is the total length of the exons of lincRNA ortholog. Manual examination of orthologous lincRNA alignments and putative orthologs suggested that approximately 5% of the alignments with the largest INDEL values were unreliable. Thus, all lincRNA alignments with INDEL >95% were removed from further analysis. Similarly, a cut-off was imposed on expression level of putative human and mouse orthologs of lincRNA. This cut-off was set at the lowest 5% of the expression levels of the 196 orthologous validated lincRNA genes (Supporting Table S1). All putative orthologs of lincRNA genes with lower expression values were discarded under the premise that these low values could represent experimental noise, i.e. the top 95% of the expression values EXP95% was used for all analyses (Table 1 and Supporting Table S1). In addition, EXP90%, 80%, 70%, 60%, 50%, 40%, 30%, 20%, 10% were calculated to compare subsets of lincRNAs expressed at different levels (Table 2). We also used different sets of expression/indel filters combined with the 5 input parameters (see Results) in different experiments (Tables 1 and 2); no substantial differences between results were found (see Discussion for details). For calculating the 5 input parameters (see Results), all the collected information was stored in an SQLite database, and after applying ORF, indel and expression thresholds, final data sets were assembled (Tables 1, 2 and Supporting Table S1).

### Maximum likelihood estimates

Using the experimentally validated sets of human and mouse lincRNAs and the assumptions described in the main text the probability of observing a particular set of *Kh*, *Km* and *Kb* for the given values of *Lh* and *Lm* is given by equation (1) in the main text. Using the Sterling's approximation for the factorial, we obtain the system of nonlinear equations for the sizes *Nh* and *Nm* of the pools and their overlap *Nb* that maximize the likelihood *P* in Eq. (1)

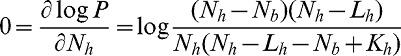
(3)

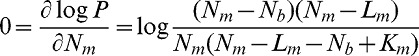
(4)

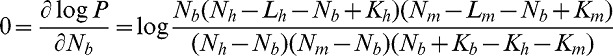
(5)Solving the system (3–5) for *Nh*, *Nm* and *Nb* we obtain Equation (2) (see main text).

The confidence region around the maximum likelihood estimate Eq. (5) is an ellipsoid in the {*Nh,Nm,Nb*} space. The directions of its axes are given by the eigenvectors of the Jacobian matrix *J* of second derivatives of log *P* and the magnitudes of the ellipsoid's axes are given by the inverse square roots of the negatives of the eigenvalues. Computing the second derivatives of log *P* and evaluating them at the maximum likelihood point, we obtain
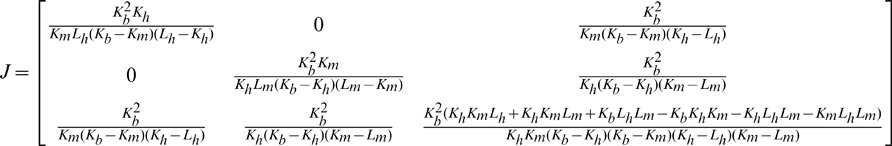
(6)


We found that the confidence ellipsoid is highly elongated, and therefore the estimates for the pool sizes are strongly correlated with each other. The analytically estimated 95% confidence intervals are shown in Table 1.

In addition, a bootstrap analysis of the lincRNA numbers was performed. For this purpose, the initial sets of human and mouse lincRNAs were randomly resampled 1000 times and the calculation of the final numbers was performed using 95% indel and expression (RPKM) levels, and all ORF thresholds. The results of bootstrap analysis are given in the Supporting Table S1. The 95% confidence intervals estimated using the boostrapping procedure (Supporting Table S1) were smaller than the analytically obtained 95% confidence intervals (Table 1), thus we used the latter as conservative estimates of the 95% confidence intervals.

## Supporting Information

Table S1 Comprehensive information on the human and mouse lincRNA sets.
(XLS)

## References

[1] Birney E, Stamatoyannopoulos JA, Dutta A, Guigo R, Gingeras TR, et al (2007) Identification and analysis of functional elements in 1% of the human genome by the ENCODE pilot project. Nature 447: 799–816. 17571346 10.1038/nature05874 PMC2212820

[2] Amaral PP, Dinger ME, Mercer TR, Mattick JS (2008) The eukaryotic genome as an RNA machine. Science 319: 1787–1789. 18369136 10.1126/science.1155472

[3] Clark MB, Amaral PP, Schlesinger FJ, Dinger ME, Taft RJ, et al (2011) The reality of pervasive transcription. PLoS Biol 9: e1000625 discussion e1001102. 21765801 10.1371/journal.pbio.1000625 PMC3134446

[4] Bernstein BE, Birney E, Dunham I, Green ED, Gunter C, et al (2012) An integrated encyclopedia of DNA elements in the human genome. Nature 489: 57–74. 22955616 10.1038/nature11247 PMC3439153

[5] Kapranov P, Cawley SE, Drenkow J, Bekiranov S, Strausberg RL, et al (2002) Large-scale transcriptional activity in chromosomes 21 and 22. Science 296: 916–919. 11988577 10.1126/science.1068597

[6] Rinn JL, Euskirchen G, Bertone P, Martone R, Luscombe NM, et al (2003) The transcriptional activity of human Chromosome 22. Genes Dev 17: 529–540. 12600945 10.1101/gad.1055203 PMC195998

[7] Bertone P, Stolc V, Royce TE, Rozowsky JS, Urban AE, et al (2004) Global identification of human transcribed sequences with genome tiling arrays. Science 306: 2242–2246. 15539566 10.1126/science.1103388

[8] Cheng J, Kapranov P, Drenkow J, Dike S, Brubaker S, et al (2005) Transcriptional maps of 10 human chromosomes at 5-nucleotide resolution. Science 308: 1149–1154. 15790807 10.1126/science.1108625

[9] Okazaki Y, Furuno M, Kasukawa T, Adachi J, Bono H, et al (2002) Analysis of the mouse transcriptome based on functional annotation of 60,770 full-length cDNAs. Nature 420: 563–573. 12466851 10.1038/nature01266

[10] Katayama S, Tomaru Y, Kasukawa T, Waki K, Nakanishi M, et al (2005) Antisense transcription in the mammalian transcriptome. Science 309: 1564–1566. 16141073 10.1126/science.1112009

[11] Carninci P, Kasukawa T, Katayama S, Gough J, Frith MC, et al (2005) The transcriptional landscape of the mammalian genome. Science 309: 1559–1563. 16141072 10.1126/science.1112014

[12] Ponjavic J, Ponting CP, Lunter G (2007) Functionality or transcriptional noise? Evidence for selection within long noncoding RNAs. Genome Res 17: 556–565. 17387145 10.1101/gr.6036807 PMC1855172

[13] Mattick JS, Makunin IV (2006) Non-coding RNA. Hum Mol Genet 15 Spec No 1: R17–29. 16651366 10.1093/hmg/ddl046

[14] Mercer TR, Dinger ME, Mattick JS (2009) Long non-coding RNAs: insights into functions. Nat Rev Genet 10: 155–159. 19188922 10.1038/nrg2521

[15] Ponting CP, Belgard TG (2010) Transcribed dark matter: meaning or myth? Hum Mol Genet 19: R162–168. 20798109 10.1093/hmg/ddq362 PMC2953743

[16] Ponting CP, Oliver PL, Reik W (2009) Evolution and functions of long noncoding RNAs. Cell 136: 629–641. 19239885 10.1016/j.cell.2009.02.006

[17] Khalil AM, Guttman M, Huarte M, Garber M, Raj A, et al (2009) Many human large intergenic noncoding RNAs associate with chromatin-modifying complexes and affect gene expression. Proc Natl Acad Sci U S A 106: 11667–11672. 19571010 10.1073/pnas.0904715106 PMC2704857

[18] van Bakel H, Nislow C, Blencowe BJ, Hughes TR (2010) Most “dark matter” transcripts are associated with known genes. PLoS Biol 8: e1000371. 20502517 10.1371/journal.pbio.1000371 PMC2872640

[19] Robinson R (2010) Dark matter transcripts: sound and fury, signifying nothing? PLoS Biol 8: e1000370. 20502697 10.1371/journal.pbio.1000370 PMC2872672

[20] Marques AC, Ponting CP (2009) Catalogues of mammalian long noncoding RNAs: modest conservation and incompleteness. Genome Biol 10: R124. 19895688 10.1186/gb-2009-10-11-r124 PMC3091318

[21] Siepel A, Bejerano G, Pedersen JS, Hinrichs AS, Hou M, et al (2005) Evolutionarily conserved elements in vertebrate, insect, worm, and yeast genomes. Genome Res 15: 1034–1050. 16024819 10.1101/gr.3715005 PMC1182216

[22] Guttman M, Amit I, Garber M, French C, Lin MF, et al (2009) Chromatin signature reveals over a thousand highly conserved large non-coding RNAs in mammals. Nature 458: 223. 19182780 10.1038/nature07672 PMC2754849

[23] Managadze D, Rogozin IB, Chernikova D, Shabalina SA, Koonin EV (2011) Negative correlation between expression level and evolutionary rate of long intergenic noncoding RNAs. Genome Biol Evol 3: 1390–1404. 22071789 10.1093/gbe/evr116 PMC3242500

[24] Huttenhofer A, Vogel J (2006) Experimental approaches to identify non-coding RNAs. Nucleic Acids Res 34: 635–646. 16436800 10.1093/nar/gkj469 PMC1351373

[25] Amaral PP, Clark MB, Gascoigne DK, Dinger ME, Mattick JS (2011) lncRNAdb: a reference database for long noncoding RNAs. Nucleic Acids Res 39: D146–151. 21112873 10.1093/nar/gkq1138 PMC3013714

[26] Liu J, Gough J, Rost B (2006) Distinguishing protein-coding from non-coding RNAs through support vector machines. PLoS Genet 2: e29. 16683024 10.1371/journal.pgen.0020029 PMC1449884

[27] Cabili MN, Trapnell C, Goff L, Koziol M, Tazon-Vega B, et al (2011) Integrative annotation of human large intergenic noncoding RNAs reveals global properties and specific subclasses. Genes Dev 25: 1915–1927. 21890647 10.1101/gad.17446611 PMC3185964

[28] Wang Z, Gerstein M, Snyder M (2009) RNA-Seq: a revolutionary tool for transcriptomics. Nat Rev Genet 10: 57–63. 19015660 10.1038/nrg2484 PMC2949280

[29] Mortazavi A, Williams BA, McCue K, Schaeffer L, Wold B (2008) Mapping and quantifying mammalian transcriptomes by RNA-Seq. Nat Methods 5: 621–628. 18516045 10.1038/nmeth.1226 PMC13303166

[30] Clamp M, Fry B, Kamal M, Xie X, Cuff J, et al (2007) Distinguishing protein-coding and noncoding genes in the human genome. Proc Natl Acad Sci U S A 104: 19428–19433. 18040051 10.1073/pnas.0709013104 PMC2148306

[31] Church DM, Goodstadt L, Hillier LW, Zody MC, Goldstein S, et al (2009) Lineage-specific biology revealed by a finished genome assembly of the mouse. PLoS Biol 7: e1000112. 19468303 10.1371/journal.pbio.1000112 PMC2680341

[32] Wilhelm BT, Marguerat S, Watt S, Schubert F, Wood V, et al (2008) Dynamic repertoire of a eukaryotic transcriptome surveyed at single-nucleotide resolution. Nature 453: 1239–1243. 18488015 10.1038/nature07002

[33] Pevzner P, Tesler G (2003) Genome rearrangements in mammalian evolution: lessons from human and mouse genomes. Genome Res 13: 37–45. 12529304 10.1101/gr.757503 PMC430962

[34] van Bakel H, Hughes TR (2009) Establishing legitimacy and function in the new transcriptome. Brief Funct Genomic Proteomic 8: 424–436. 19833698 10.1093/bfgp/elp037

[35] Ebisuya M, Yamamoto T, Nakajima M, Nishida E (2008) Ripples from neighbouring transcription. Nat Cell Biol 10: 1106–1113. 19160492 10.1038/ncb1771

[36] Mercer TR, Wilhelm D, Dinger ME, Solda G, Korbie DJ, et al (2011) Expression of distinct RNAs from 3′ untranslated regions. Nucleic Acids Res 39: 2393–2403. 21075793 10.1093/nar/gkq1158 PMC3064787

[37] Brosius J, Tiedge H (2004) RNomenclature. RNA Biol 1: 81–83. 17179746 10.4161/rna.1.2.1228

[38] Dinger ME, Pang KC, Mercer TR, Mattick JS (2008) Differentiating protein-coding and noncoding RNA: challenges and ambiguities. PLoS Comput Biol 4: e1000176. 19043537 10.1371/journal.pcbi.1000176 PMC2518207

[39] Makalowska I, Rogozin IB, Makalowski W (2010) Genome evolution. Adv Bioinformatics 2010: 643701. 21904546 10.1155/2010/643701 PMC3166573

[40] Carvunis AR, Rolland T, Wapinski I, Calderwood MA, Yildirim MA, et al (2012) Proto-genes and de novo gene birth. Nature 487: 370–374. 22722833 10.1038/nature11184 PMC3401362

[41] Churbanov A, Rogozin IB, Babenko VN, Ali H, Koonin EV (2005) Evolutionary conservation suggests a regulatory function of AUG triplets in 5′-UTRs of eukaryotic genes. Nucleic Acids Res 33: 5512–5520. 16186132 10.1093/nar/gki847 PMC1236974

[42] Neafsey DE, Galagan JE (2007) Dual modes of natural selection on upstream open reading frames. Mol Biol Evol 24: 1744–1751. 17494029 10.1093/molbev/msm093

[43] Wen Y, Liu Y, Xu Y, Zhao Y, Hua R, et al (2009) Loss-of-function mutations of an inhibitory upstream ORF in the human hairless transcript cause Marie Unna hereditary hypotrichosis. Nat Genet 41: 228–233. 19122663 10.1038/ng.276

[44] Calvo SE, Pagliarini DJ, Mootha VK (2009) Upstream open reading frames cause widespread reduction of protein expression and are polymorphic among humans. Proc Natl Acad Sci U S A 106: 7507–7512. 19372376 10.1073/pnas.0810916106 PMC2669787

[45] Meijer HA, Thomas AA (2002) Control of eukaryotic protein synthesis by upstream open reading frames in the 5′-untranslated region of an mRNA. Biochem J 367: 1–11. 12117416 10.1042/BJ20011706 PMC1222879

[46] Ingolia NT, Ghaemmaghami S, Newman JR, Weissman JS (2009) Genome-wide analysis in vivo of translation with nucleotide resolution using ribosome profiling. Science 324: 218–223. 19213877 10.1126/science.1168978 PMC2746483

[47] Ingolia NT, Lareau LF, Weissman JS (2011) Ribosome profiling of mouse embryonic stem cells reveals the complexity and dynamics of mammalian proteomes. Cell 147: 789–802. 22056041 10.1016/j.cell.2011.10.002 PMC3225288

[48] Banfai B, Jia H, Khatun J, Wood E, Risk B, et al (2012) Long noncoding RNAs are rarely translated in two human cell lines. Genome Res 22: 1646–1657. 22955977 10.1101/gr.134767.111 PMC3431482

[49] Brosius J (2005) Waste not, want not–transcript excess in multicellular eukaryotes. Trends Genet 21: 287–288. 15851065 10.1016/j.tig.2005.02.014

[50] Huttenhofer A, Schattner P, Polacek N (2005) Non-coding RNAs: hope or hype? Trends Genet 21: 289–297. 15851066 10.1016/j.tig.2005.03.007

[51] Waterston RH, Lindblad-Toh K, Birney E, Rogers J, Abril JF, et al (2002) Initial sequencing and comparative analysis of the mouse genome. Nature 420: 520–562. 12466850 10.1038/nature01262

[52] Ogurtsov AY, Sunyaev S, Kondrashov AS (2004) Indel-based evolutionary distance and mouse-human divergence. Genome Res 14: 1610–1616. 15289479 10.1101/gr.2450504 PMC509270

[53] Lunter G, Ponting CP, Hein J (2006) Genome-wide identification of human functional DNA using a neutral indel model. PLoS Comput Biol 2: e5. 16410828 10.1371/journal.pcbi.0020005 PMC1326222

[54] Nesterova TB, Slobodyanyuk SY, Elisaphenko EA, Shevchenko AI, Johnston C, et al (2001) Characterization of the genomic Xist locus in rodents reveals conservation of overall gene structure and tandem repeats but rapid evolution of unique sequence. Genome Res 11: 833–849. 11337478 10.1101/gr.174901 PMC311126

[55] Brannan CI, Dees EC, Ingram RS, Tilghman SM (1990) The product of the H19 gene may function as an RNA. Mol Cell Biol 10: 28–36. 1688465 10.1128/mcb.10.1.28-36.1990 PMC360709

[56] Karolchik D, Hinrichs AS, Furey TS, Roskin KM, Sugnet CW, et al (2004) The UCSC Table Browser data retrieval tool. Nucleic Acids Res 32: D493–496. 14681465 10.1093/nar/gkh103 PMC308837

[57] Quinlan AR, Hall IM (2010) BEDTools: a flexible suite of utilities for comparing genomic features. Bioinformatics 26: 841–842. 20110278 10.1093/bioinformatics/btq033 PMC2832824

[58] ENCODE Project Consortium (2011) A user's guide to the encyclopedia of DNA elements (ENCODE). PLoS Biol 9: e1001046. 21526222 10.1371/journal.pbio.1001046 PMC3079585

[59] Trapnell C, Roberts A, Goff L, Pertea G, Kim D, et al (2012) Differential gene and transcript expression analysis of RNA-seq experiments with TopHat and Cufflinks. Nat Protoc 7: 562–578. 22383036 10.1038/nprot.2012.016 PMC3334321

[60] Rogozin IB, Kochetov AV, Kondrashov FA, Koonin EV, Milanesi L (2001) Presence of ATG triplets in 5′ untranslated regions of eukaryotic cDNAs correlates with a ‘weak’ context of the start codon. Bioinformatics 17: 890–900. 11673233 10.1093/bioinformatics/17.10.890

[61] Milanesi L, D'Angelo D, Rogozin IB (1999) GeneBuilder: interactive in silico prediction of gene structure. Bioinformatics 15: 612–621. 10487869 10.1093/bioinformatics/15.7.612

[62] Hinrichs AS, Karolchik D, Baertsch R, Barber GP, Bejerano G, et al (2006) The UCSC Genome Browser Database: update 2006. Nucleic Acids Res 34: D590–598. 16381938 10.1093/nar/gkj144 PMC1347506

[63] International Human Genome Sequencing Consortium (2004) Finishing the euchromatic sequence of the human genome. Nature 431: 931–945. 15496913 10.1038/nature03001

